# Informed or misinformed consent and use of modified texture diets in dysphagia

**DOI:** 10.1186/s12910-023-00885-1

**Published:** 2023-02-07

**Authors:** Shaun T. O’Keeffe, Paula Leslie, Tracy Lazenby-Paterson, Arlene McCurtin, Lindsey Collins, Aoife Murray, Alison Smith, Siofra Mulkerrin, SPARC (Swallow Perspectives, Advocacy and Research Collective)

**Affiliations:** 1 Department of Geriatric Medicine, Galway University Hospitals, Newcastle Rd, Galway, Ireland; 2 Northern Medical Physics and Clinical Engineering, Newcastle upon Tyne Hospitals NHS Trust, Newcastle upon Tyne, UK; 3 NHS Lothian Community Learning Disability Service, Leith Community Treatment Centre, Edinburgh, UK; 4 School of Allied Health, University of Limerick, Limerick, Ireland; 5 Health Implementation Science and Technology Research Group, Health Research Institute, University of Limerick, Limerick, Ireland; 6 Centre for Applied Dementia StudiesFaculty of Health Studies, University of Bradford, Bradford, UK; 7 School of Medicine, National University of Ireland Galway, Galway, Ireland; 8 Pharmacy and Medicines Optimisation Team, Herts Valleys Clinical Commissioning Group, Hemel Hempstead, UK; 9 Department of Speech and Language Therapy, Addenbrooke’s Hospital, Cambridge, England, UK

**Keywords:** Modified texture diet, Dysphagia, Informed consent, Shared decision making, Duty of care, Quality of life, Ethics

## Abstract

**Background:**

Use of modified texture diets—thickening of liquids and modifying the texture of foods—in the hope of preventing aspiration, pneumonia and choking, has become central to the current management of dysphagia. The effectiveness of this intervention has been questioned. We examine requirements for a valid informed consent process for this approach and whether the need for informed consent for this treatment is always understood or applied by practitioners.

**Main text:**

Valid informed consent requires provision of accurate and balanced information, and that agreement is given freely by someone who knows they have a choice. Current evidence, including surveys of practitioners and patients in different settings, suggests that practice in this area is often inadequate. This may be due to patients’ communication difficulties but also poor communication—and no real attempt to obtain consent—by practitioners before people are ‘put on’ modified texture diets. Even where discussion occurs, recommendations may be influenced by professional misconceptions about the efficacy of this treatment, which in turn may poison the well for the informed consent process. Patients cannot make appropriate decisions for themselves if the information provided is flawed and unbalanced. The voluntariness of patients’ decisions is also questionable if they are told ‘you must’, when ‘you might consider’ is more appropriate. Where the decision-making capacity of patients is in question, inappropriate judgements and recommendations may be made by substitute decision makers and courts unless based on accurate information.

**Conclusion:**

Research is required to examine the informed consent processes in different settings, but there is ample reason to suggest that current practice in this area is suboptimal. Staff need to reflect on their current practice regarding use of modified texture diets with an awareness of the current evidence and through the ‘lens’ of informed consent. Education is required for staff to clarify the importance of, and requirements for, valid informed consent and for decision making that reflects people’s preferences and values.

## Background

It is a general legal and ethical principle that valid consent must be obtained for healthcare interventions and treatments [1–3]. This principle protects the right of people to determine what happens to their own bodies and guides ethical practice of health care. For consent to be valid, it must be informed and voluntary and the person consenting must have the capacity to make the decision. “Informed” means the person must be given sufficient information in a way they can understand about what the treatment involves, including the potential benefits and harms, whether there are reasonable alternative treatments, and what will happen if treatment does not go ahead. “Voluntary” means the decision to consent or to refuse treatment must be made freely by the person and must not be due to coercion—under undue pressure imposed by others—such that the person believes there is no alternative but to ‘consent’. Having “capacity” means the person can understand, retain, use and weigh up the information relevant to their decision—such as the options available to them and the likely consequences of the choices they make—and can communicate their decision in some way [4–6].

Oropharyngeal dysphagia, causing difficulties with eating, drinking and swallowing is a common and distressing problem in older people and in those with neurological and neurodegenerative diseases [7, 8]. Those who have such difficulty have a greater risk of fatal and near-fatal asphyxiation due to choking on food [9, 10]. The likelihood of developing pneumonia is also greater in those with dysphagia, although dysphagia is not the most important risk factor and is not sufficient of itself to cause pneumonia [11]. Modified texture diets—by which we mean both thickening of liquids and modifying the texture of foods—in the hope of improving swallow safety and control and preventing aspiration, pneumonia and choking, have become central to the current management of dysphagia [10, 12, 13].

There is now an extensive literature reviewing the effects of modified texture diets and which acknowledges that the evidence base supporting the purported benefits of this approach is limited. There is also potential for such diets to cause harm including poorer hydration and nutrition, a significant adverse impact on quality of life and increased social isolation [14]. We, and others, have argued that in current clinical practice this approach is excessively employed and that many people with dysphagia are being limited to unnecessarily restrictive diets [14–20].

If practitioners choose to recommend modified texture diets to people with dysphagia, this is a healthcare treatment and thus requires informed consent. In this narrative paper, we examine what is needed for a valid informed consent process for use of modified texture diets in the context of the known empirical data regarding the benefits and harms associated with this intervention. We emphasise the need to convey the uncertainties regarding the balance of benefit and harm so that people can make informed and voluntary choices. We suggest that there is evidence that the need for informed consent is not always understood or applied by practitioners. Furthermore, we contend that concepts such as *shared decision making* and *duty of care* are sometimes incorrectly invoked as alternatives to individual consent in current practice.

## Main text

### Is informed consent required for use of modified texture diets?

Modifying the texture of food is not always a healthcare treatment. Eating and drinking are basic human needs (and pleasures), and most people enjoy a great variety of food textures, sizes, and liquid consistencies as part of a normal diet. Providing and preparing food and drink, even in a healthcare setting, or assisting someone to eat and drink are not primarily healthcare interventions. It is not always necessary to ‘medicalise’ the modification of food textures to make them easier to swallow: offering to chop up food for a person who cannot do it easily themselves, or offering gravy if a dish looks dry, are often simply matters of courtesy and kindness. The same is true of much of the common-sense advice regarding how to eat, even if provided by healthcare professionals: advice like ‘don’t gobble your food’, ‘don’t talk while you’re eating’ and ‘don’t drink too quickly’ do not ‘belong’ to professionals.

The same arguments cannot be made when a modified texture diet requires adding commercial thickeners to liquids or significantly altering the texture of food for healthcare purposes rather than for culinary pleasure. Ultimately, implementation of such recommendations (or ‘prescriptions’) by healthcare professionals are unequivocally healthcare treatments and thus require informed consent.

### Informed consent and shared decision-making

The term shared decision making (SDM) was first defined by the US President’s Commission for the Study of Ethical Research in a 1983 report focused on informed consent [21]. The report criticised the traditional medical model for informed consent as viewing communication primarily about the giving and receiving of information rather than also needing clarification of the values and goals of the person. Consenting in this way “*connotes passivity and acceptance, not active engagement and participation*” [22, p. 134]. It is true, as Makoul and Clayman—who identified 31 separate concepts used to explain SDM in a review of the literature—put it “*SDM has been variably, and often loosely, defined*” [23, p. 301].

The concept and process of SDM was developed to promote better mutual communication and understanding, to facilitate a patient-centred rather than a professional-centred standard for informed consent and to ensure that decisions integrated the best evidence with the person’s values and preferences [21]. Ultimately, SDM is a way to enhance the quality of informed consent, not to replace it [24]. Laws and professional guidelines in many jurisdictions increasingly use the language of SDM in guidance and rules about informed consent [1, 25]. Decision aids—interventions that help patients by supporting congruence between decisions and personal values—can be useful in promoting SDM and a more active role for patients in decision making. especially for treatments that do not yet have high‐quality evidence [26].

The ‘shared’ in SDM should not be interpreted literally: SDM does not imply joint decision making (where both parties must agree) or any reduction or dilution of the patient’s decision-making authority. The essence of SDM is “*one of profound respect for the right of every patient to chart his or her own course*” [27, p. 55]. However, this is not clear from use of the term in some of the dysphagia literature. For example, Kaizer et al.’s statement that “[d]*ivergent views regarding diet modification can strain the therapeutic relationship between patients/families and the treating team, and hamper efforts toward shared decision-making*” implies that decisions should be made jointly in SDM [28, p. 82]. Further, the emphasis on sharing with the patient and ensuring their voice is heard may be replaced by an emphasis on sharing with and within the healthcare team [29].

Another misuse of the concept of SDM is to see it as a tool for handling “non-compliance” in those with dysphagia [13, 28, 30]. For example, if a patient wishes to make a choice that a clinician feels is unduly risky “*it is consistent with* [SDM] *for health-care professionals to begin* [our emphasis] *to clarify the implications of a decision for the patient and attempt to understand the issue from the patient’s perspective*” [28, p. 83]. The ‘begin’ here suggests that the initial approach when proposing modified texture diets in dysphagia is and should be simple education of the person, ideally leading to their acceptance of the clinical recommendation, and that SDM is an ‘add-on’ involving education and even repeated re-education of such patients so they can come to agree to what is recommended for them. In fact, SDM requires incorporation of the person’s preferences from the outset [24].

### Informed consent and duty of care

Healthcare professionals owe a duty of care to their patients which is the basis of laws of negligence in health care. Professionals are required to apply *“the degree of care and skill which is expected of the average practitioner in the class to which he belongs, acting in the same or similar circumstances”* to avoid a reasonably foreseeable injury [31, 32]. It has been argued that when patients won’t accept a recommended diet modification, there is a conflict for healthcare professionals between their desire to respect a patient’s autonomy of choice (and informed refusal), and their duty of care and professional and legal obligations to try to avoid harm [13, 28].

When considering the alleged conflict between duty of care and respect for informed consent we should remember that seeking informed consent is part of the duty of care for professionals and is embedded in professional standards [33]. A duty of care is a source of obligation for healthcare professionals which “*does not provide any power to those who bear it*” [34, p. 2]. It reinforces the responsibility to respect informed consent and informed refusal rather than releasing professionals from this responsibility. It does not provide a right or obligation to impose care or treatment [35]. This is not always understood; for example, an Australian study noted healthcare professionals treating mental health disorders misused duty of care as a justification for coercive practices in those refusing treatment [34].

We accept that there are limited situations where genuine conflicts arise between a desire to avoid what seems very likely serious harm and to respect a person’s choice and that these situations may be a source of distress and anxiety for staff. This may arise particularly if staff are directly involved in providing a non-recommended diet to a person. We have argued that there is a limit to what patients can demand of staff [36, pp. 68–69)]. In our view:

“*If staff are responsible for feeding someone who is at high risk of choking to death, they would be perfectly entitled to say: ‘No. I won’t administer food of a size or at a speed that will clearly be dangerous for you. I will cut up that large piece of steak and only give you the next piece when you’ve swallowed this one’*”[36].

This does not serve as a general argument for refusing to accept a person’s choices regarding texture modified diets in the interests of ‘safety’—a multidimensional concept that may be misinterpreted as avoidance of all possible adverse events [37]. This is even more true where there is uncertainty about the potential for harm and of benefit, as is the case for texture modified diets in general.

The concept of duty of care is also relevant in situations where patients are kept nil by mouth. Providing food and fluid in a healthcare setting is basic care. For example, an investigation into the death of a patient with dementia and dysphagia suggested that failure to provide food and fluid for a prolonged period for fear of aspiration, and despite her distress at this approach, was “*extraordinary and unacceptable*” and represented a failure of the duty of care owed to her [38, p. 15].

### Who can seek informed consent for use of modified texture diets?

As a general principle, it is the healthcare professional who is proposing a particular intervention who is responsible for obtaining informed consent. What is important is not the professional group per se, but that those who provide information and seek consent have sufficient knowledge themselves of the information that needs to be conveyed and the requirements for informed consent.

In the hospital setting, it is often speech and language therapists (SLTs) who make recommendations and are thus responsible for seeking consent for modified texture diets to be provided. The need for informed consent extends to the conduct of clinical swallow examinations and instrumental assessments such as videofluoroscopic or flexible endoscopic procedures that may guide recommendations to use modified texture diets. Arguably consent for swallow assessments also requires that people can weigh up the potential harms and benefits of the outcome including possible predicted treatments like modified texture foods [39–41].

Some decisions are complex, and there may be a variety of other disciplines in a multidisciplinary team supporting those who eat, drink and swallow with difficulty. While having a variety of views is generally helpful, it is important that it does not lead to a diffusion of the responsibility for seeking informed consent, with no professional being required to take personal responsibility for communicating with the patient and eliciting their views and choice [36].

In residential care settings, where there may not be ready access to SLTs, other professionals such as nurses may initiate use of modified texture diets without other professional input [42]. Those professionals have taken on the responsibility for seeking consent (although we argue later that they may be unaware of this responsibility or may lack the skills and knowledge to fulfil this role).

### One-off or repeated consent for modified texture diets?

This raises two important issues. Firstly, informed consent is often a process involving repeated discussions rather than a single discrete decision. This is particularly important when the clinical situation changes or evolves. In clinical practice there are often natural decision points arising over time when consent discussions should recur as relevant information and the balance of potential benefits and harms for interventions change for the person. Although the nature and scope of the dietary recommendations may change, it is important that the patient remains the ultimate decision maker throughout.

Consideration of modified texture diets in those with dysphagia after an acute stroke provides a useful example of how recommendations and discussions may evolve with time:

- In the acute phase, a patient is often frightened and upset at what has happened and effective communication may be impaired even in the absence of aphasia; dysphagia may resolve and is subject to ongoing assessment; and enhanced monitoring means the risks of undernutrition and underhydration are less (although not absent) [43].
- In the rehabilitation setting, the nature of the impairment is likely to have changed; a better sense of the likely long-term prognosis is emerging, and there is greater opportunity to elicit the patient’s preferences and goals of care.
- Discharge home or to residential care represents a major decision point for those who still have significant dysphagia. Even if there is hope for more improvement, recommendations for modified texture diets, if made, often represent a long-term strategy.

The second important point is that having a genuine choice always means that patients can refuse or withdraw consent at any stage: for example, they may consent to surgery, and sign the forms, and then change their mind and refuse the operation, or they may initially agree to take a medication but then discontinue it because it makes them nauseous.

This is particularly relevant with regard to modified texture diets because they often end up as a long-term measure, with insufficient follow up or review, and people eat and drink many times a day. A patient who agrees with a recommendation to take a particular modified texture diet is not entering a ‘binding covenant’ that can only be broken by mutual agreement. Professionals should look to review their previous advice for current appropriateness. It is also important that informal caregivers, families and healthcare workers who were not involved in the initial consent process, for example staff in residential care facilities or family doctors, are aware that people have such choices and can change their minds. It is unfortunate that regulatory guidance for healthcare professionals is sometimes ambiguous on this point; for example, the English Care Quality Commission guidance states: “*Where a person is assessed as needing a specific diet, this must* [our emphasis] *be provided in line with that assessment*” [44].

### What are the requirements for valid informed consent regarding modified texture diets?

#### Information provision

Informed consent requires provision of relevant information in a way that people can understand. This should address the potential harms, benefits and address any uncertainties regarding treatment options. There are some important general principles.

- There is no requirement that patients become experts regarding the intervention, as the phrase “fully informed consent” may suggest [45].
- The amount of information to be shared depends in part on the seriousness and intrusiveness of the intervention. The stakes are high regarding use of modified texture diets: the intervention is intrusive, and can be enduring in practice, and both the purported benefits and harms are significant.
- It is never acceptable for someone seeking consent to focus on the potential benefits of an intervention and fail to discuss potential adverse effects for the person. Just as a cardiologist prescribing aspirin is obliged to explain the risk of serious gastrointestinal side effects, an SLT is required to discuss the possible impact of thickened fluids, for example, on hydration and therefore renal function.
- The harms to be discussed are those that “*in the circumstances of the particular case, a reasonable person in the patient’s position*” would be likely to consider important [46]. An adverse effect on quality of life and enjoyment of eating and of eating out is an important consequence of the use of modified texture diets and is as important an issue for discussion with patients as any physical effects.

Ultimately, the information provided must be accurate and balanced. This requires consideration of the quality of evidence that an intervention will be successful in achieving clinically meaningful endpoints that are important to a patient. There is no robust evidence at present to suggest that modified texture diets benefit adults with dysphagia by preventing pneumonia and its consequences. As the authors of a recent textbook on dysphagia noted: “*Simply stated, we have no strong guidelines to “match” a diet level or degree of thickened liquid to a patient based on clinical or imaging studies as currently engaged*” [47, p. 425].

These are not (or should not be seen as) contentious statements: they reflect the current state of knowledge and are supported by multiple reviews of the topic [12, 14, 17, 48–53]. It is true that “absence of evidence is not evidence of absence”. Thickened liquids, for example, might reduce, but not eliminate, the volume of aspirate for some but have not been shown to date to reduce the risk of pneumonia [14]. Indeed, the only large randomised clinical trial suggested that very thick fluids may increase the risk and severity of pneumonia [54]. If modified texture diets are proposed, patients need to know of this uncertainty in the evidence to make their own informed choice (Table 1).

**Table 1:** Summary of potential benefits and harms of modified texture diets

Intervention	Potential benefit	Potential harm	Comments/evidence
Thickened liquids	Reduced penetration–aspiration with liquids from thin to very thick end of the viscosity continuum [55]. Reduced penetration–aspiration might mean less risk of pneumonia with TL	Increased risk of post-swallow pharyngeal residue for liquids with higher viscosities. Increased residue and reduced cilial clearance with TL might mean more risk of pneumonia	There is no evidence to suggest TL reduce pneumonia [50]. There is some limited evidence that very thick liquids lead to more and to more severe pneumonia [54]. Animal studies suggest that aspiration of TL causes more lung inflammation than aspiration of thin liquids [56]
	Easier to control swallowing with TL may mean less distress and coughing when drinking	TL are less thirst quenching and pleasant to drink [57]	The balance of evidence is that TL have an adverse impact on QOL, and many people will not accept them as a result [58]
		Reduced fluid intake and greater risk of dehydration and renal impairment	Biochemical indices showing underhydration are common in those receiving TL [43]
		Reduced bioavailability of some medications [59]	Particular concern for drugs with a narrow window between toxicity and benefit
		Lack of follow-up assessment regarding long-term clinical/QOL impact	Lack of resources especially in residential care facilities [14]
Modified food	Reduced risk of asphyxiation and death from large bolus obstruction		Cutting food to bite sized chunks will reduce risk of asphyxiation and death [13]
		Reduced food intake and increased risk of undernutrition	A reduction in food intake is common with MF [60]. Although changes in the diets prescribed can mitigate to some degree [61], pureed diets contribute to a high prevalence of malnutrition in those with dysphagia and often have poorer calorie, protein and micronutrient content than regular diets
		Reduced quality of life and enjoyment of eating	MF have an adverse impact on quality of life. The more modified the food texture, the worse the quality of life [58]
		Lack of follow-up assessment regarding long-term clinical/QOL impact	Lack of resources especially in residential care facilities [14]

*TL* Thickened liquids, *MF* Modified foods, *QOL* Quality of life

#### Voluntariness of decisions regarding modified texture diets

For consent to be valid it must be given freely and voluntarily. People must be supported to understand that they are the decision makers and have choices, including to refuse to consent. Coercion is not simply physical force or overt threats. The words used, the tone of voice, and body language can be coercive if they lead to a lack of, or a conditionality of, choice. Potentially coercive language includes terms like ‘you must…’ or ‘you are not allowed…’ or ‘you cannot do that unless…’, or ‘if you choose that option there is nothing else I can offer’.

‘You must’ and related terms are not to be used lightly: they suggest a high degree of certainty about what is best for the person, and this is particularly problematic if used regarding a treatment like modified texture diets where the evidence base is limited. It is important to distinguish the limitations of choice imposed by illness from those imposed by others. If someone has type 1 diabetes mellitus, for example, they really ‘must’ take insulin if they have any regard for their life or health, and it is necessary for a professional to emphasize that failure to take insulin will inevitably lead to death. In dysphagia management, a professional might be justified in, for example, strongly recommending cutting food into bite sized chunks if someone is known to be at high risk for asphyxiation. A similarly strong recommendation regarding modified texture diets is not justified on current evidence if the aim is to prevent death from pneumonia.

#### Having capacity to make decisions regarding modified texture diets

The presumption of capacity is as fundamental to decision-making as the presumption of innocence in criminal trials [62]. The person “*has to ‘prove’ nothing*” [63, p. 2], and the burden of proving a *lack* of capacity to take a specific decision always lies upon the professional who is challenging capacity. One pervasive and damaging form of ageism is that advanced age of itself leads in effect to a presumption of lack of capacity and to a paternalistic protectionism by professionals.

The presumption of capacity is open to challenge if there is sufficient evidence or reason to do so. There are situations in the management of dysphagia where the actions of a person are so obviously foolish and reckless that they may give rise to legitimate concerns about whether they have decision making capacity. This might include, for example, someone with a history of near fatal choking who is eating food portions that are obviously dangerous. This does not imply that we must question a patient’s choice just because it may involve some chance of a poor outcome or is contrary to the advice of professionals. It seems entirely rational for somebody to emphasise their quality of life over all other considerations if they find a particular diet unacceptable to them [57, 58]. This is even more so if the evidence of benefit for that diet is flimsy (and they are informed of this).

### Are the requirements for valid informed consent regarding modified texture diets met in practice?

There have been no large-scale studies, to our knowledge, on whether and how informed consent for modified texture diets is obtained in routine clinical practice in different settings. Nevertheless, we suggest in the following sections of the paper that there is good reason to suspect that current practice in this regard is inadequate (although it is likely that there is better and worse practice among individual practitioners).

#### The need for informed consent may not always be recognized

When SLTs and others have discussions with patients about modified texture diets, it is unclear whether they recognize the necessity of satisfying the formal requirements of informed consent as part of this process. Askren and Leslie noted regarding SLTs that many struggle with the patient’s right to decline recommended interventions and: “*many still feel uncomfortable with the informed consent process… In fact, many* [SLTs] *do not even acknowledge these areas as aspects innate to clinical practice*” [64, p. 163]. An influential European position paper was unhelpfully ambiguous on the topic, noting: “*Yet, there are some forms of care that seem so self-evident that one would hardly consider obtaining* [informed consent]” [65, p. 1417].

Evidence from studies asking patients and their families about the experience of modified texture diets—and the language used of being “placed on” such diets—strongly suggests that patients are sometimes not given a choice at all [42, 66–69]. In a recent study of 14 Irish patients given thickened liquids after a stroke, 13 reported not being involved in the decision to start this treatment with comments including “It came automatically”, “Somebody gave it to me” and “Nobody told me anything” [67].

Effective communication (and recollection of discussions) may be difficult in the early days after an acute stroke. However, a similar picture has been reported in studies of modified texture diets use in residential care facilities, with a lack of concern for individual preferences, ‘a blanket provision’ of modified texture diets in some homes, and comments from residents such as “I was horrified when I heard they were going to put me on a modified diet” and “the fact that you don’t get to say what you’re going to have is a huge thing” [68, 69].

#### Is the information provided about modified texture diets accurate and balanced?

Several authors have commented on a disconnect between the limited evidence base and the beliefs and practices of professionals with regard to thickened liquids and texture modified foods [14, 17, 19], and it seems in our view inevitable that such beliefs will influence how SLTs and other staff members communicate with patients about their options.

Surveys of practicing SLTs have found a strong consensus, based primarily on therapists’ training and experience and “safety-based reasoning”—in effect often a primarily defensive practice—rather than research evidence, supporting the use of modified texture diets in dysphagia [70–72]. A study of stroke clinical practice guidelines developed by expert groups of clinicians regarding the use of thickened liquids to prevent aspiration noted “*the misappropriation of evidence, non-use of recent evidence, limited use of a range of evidence, and the failure to clearly report the state of the evidence*” when recommending thickened liquids [71, p. 13].

Recent alarming reports from the United States (and it seems unlikely to occur only in the US) suggest that nurse-initiated dietary restrictions are common and problematic. A survey of 135 SLTs in the US found that 95% had encountered a practice by nurses to ‘downgrade’ dysphagia diets—that is, to introduce a more restrictive diet—without consulting SLTs [73]. The pervasive nature of this practice was confirmed by a survey of 298 practicing nurses and student nurses in the same country [74]. More than two thirds were willing to downgrade diets without an SLT opinion, whereas only a quarter would make a modified texture diet more liberal without such advice.

Very few respondents in the nursing survey strongly agreed they had adequate formal education or training with respect to dysphagia [74]. Such education is essential if one is to be professionally competent to discuss interventions and to seek the relevant informed consent. It is inadequate and misleading, for example, to hold discussions with patients about thickened liquids based on a simplistic belief that ‘thicker is safer’ in those who cough on thin fluids and without an awareness of the potential for more silent aspiration and poorer hydration [75, 76].

#### Do patients know they have a choice and are their decisions voluntary?

Reports that fewer outpatients than inpatients follow SLT recommendations regarding modified texture diets suggest that, while those living in the community can exercise their own choice, those in hospital or in nursing homes represent essentially a captive audience who cannot make their own choices [77]. Similarly, reports about people ‘cheating’ or ‘sneaking’ their preferred fluids and foods do not suggest freedom of choice [68, 69].

Half to two thirds of people with eating, drinking, and swallowing difficulties do not follow professional recommendations to take a modified texture diet [78–80]. “Compliance” language—for example “*[n]oncompliance with* [SLT] *recommendations is a serious and continuing problem within the profession*” [78, p. 30]—is often used when discussing this issue. Compliance suggests a passive behaviour by patients where they obey orders, and where non-compliance is seen as deviant, unhelpful and undesirable [22]. This language, and the attitude it reflects, is not consistent with informed consent or with an appropriate respect for positive and active involvement by people where they exercise their legal right in making their decision whether or not to accept professional recommendations.

Many people do accept and follow professional recommendations regarding modified texture diets. However, if told honestly of the limits of the evidence base supporting this practice, and of the possibility of harm and if told that the choice is theirs to make, it seems to us intuitively unlikely that many would agree to anything other than a brief trial.

#### Decision-making capacity and use of modified texture diets

The fact someone may make what looks to others to be an unwise decision is not sufficient reason to treat a person as lacking the capacity to make that decision. However, many policies regarding ‘risk feeding’ include the premise that not accepting a recommended modified texture diet may be such a risky and potentially unwise thing to do that a capacity assessment is required before a person can be ‘allowed’ to make that choice for themselves [29, 36].

Where decisional capacity to make dietary decisions is in question, misconceptions about the strength of evidence for modified texture diets may affect the conclusions of capacity assessors regarding capacity.

- If a patient refuses a recommended diet, staff may believe that this is prima facie a dangerous and potentially irrational choice that should trigger a capacity assessment that may remove the person’s decision-making authority.
- Any capacity assessment performed will be based on false premises, and thus flawed, if the information that people are supposed to understand and to use and weigh in reaching their decision is inaccurate or unbalanced.
- It is important that the information’bar’ when assessing patients is not placed too high: only essential information about the main pros and cons of different options need be understood by patients.

A significant proportion of those with dysphagia, particularly among those in long-term care facilities, may indeed lack capacity to make decisions about modified texture diets for themselves, and substitute or proxy decision makers will be needed to decide and to give or to refuse consent to this treatment on their behalf [52]. The same informed consent principles apply in this situation: such decision makers need accurate information to determine what is in the best interests of the person. If told that modified texture diets are critical to preventing pneumonia, they will inevitably give this great weight in making decisions in the best interests of the person who lacks capacity to decide for themselves, and the incapacitated person may be condemned to an unduly restrictive diet.

Furthermore, people who lack capacity to make decisions about their diet will still know what they like and do not like, even if this is expressed non-verbally such as by turning their head away or taking food from someone else’s plate. Substitute decision makers need to be informed if modified texture diets are leading to distress or reduced intake or fluid or food as it may influence the decisions they make for the person.

#### Is the need for modified texture diets and informed consent for their use reviewed?

Modified texture diets may be an appropriate short-term treatment, used in conjunction with rehabilitation strategies, in stroke and other settings [81]. However, in a large survey of American SLTs only 20% reported follow-up beyond 2 weeks after swallowing therapy, and lack of follow-up was most common for those working in acute or rehabilitation settings [82]. Local factors and service availability may impact opportunities for review of diets. Responsible professional should acknowledge and discuss such limitations with patients, who can incorporate this information when making their (initial and interim—because of course they can change mind at any time) decisions.

## Conclusion

Use of modified texture diets in the hope of preventing poor outcomes remains central to the current management of dysphagia. This is despite the lack of good evidence of benefit and the real potential for harm including a significant adverse impact on quality of life. As a healthcare intervention, use of modified texture diets for those who eat, drink and swallow with difficulty requires their informed consent (Table 2).

**Table 2:** Summary of recommended approach to informed consent for modified texture diets (MTDs)

**General approach**
As with other healthcare interventions, use of MTDs requires informed consent
The person’s values and preferences should be elicited and reflected in all discussions
Staff who provide information and seek consent must have sufficient knowledge themselves of the information that needs to be conveyed and the requirements for informed consent
**Information provision**
The information provided must be accurate and balanced, and this requires consideration of the quality of evidence that MTDs will be successful in achieving clinically meaningful endpoints that are important to a patient, and of the uncertainty regarding benefit
It is not acceptable to focus on the potential benefits and downplay the potential harms from MTDs
An adverse effect on quality of life and enjoyment of eating and drinking is as important an issue for discussion with patients as any physical effects of MTDs
Patients cannot make appropriate decisions for themselves if the information that they are given by professionals is flawed and unbalanced
**Voluntariness of decisions regarding modified texture diets**
For consent to be valid it must be given freely and voluntarily
People must be supported to understand that they are the decision makers and can make their own choices, including the choice to refuse to consent to MTDs
The voluntariness of patients’ decisions is questionable if they are told ‘you must’ when ‘you might consider’ is more appropriate
**Having capacity to make decisions regarding modified texture diets**
The burden of proving a lack of capacity to take a specific decision always lies upon the professional who is challenging capacity
Unless based on accurate and balanced information, inappropriate judgements and recommendations may be made by substitute decision makers
**Improvements needed**
Research is required to examine in greater detail current informed consent processes in different settings
There is a need for staff to reflect on their current practice regarding use of MTDs with an awareness of the currently available evidence and through the ‘lens’ of informed consent
A significant change in practice is needed in those residential care settings where there is insufficient access to SLTs and where staff may recommend or even impose MTDs without an adequate understanding of the issues involved
Professional bodies and guidelines regarding management of dysphagia should be clear about the need for informed consent before use of MTDs is advised

The acknowledged paucity of evidence supporting modified texture diets should reduce the frequency and enthusiasm with which practitioners recommend this approach. The care needed with communication is all the greater when, as with use of modified texture diets, there are uncertainties about the balance between benefit and harm. Good communication between practitioners and their patients is not solely about giving information: it requires an exploration of, and incorporation of, the person’s values and goals. This is the true meaning of shared decision making, which is an enhancement of, and not an alternative to, informed consent. Consideration of the person’s values is even more important when an intervention, like use of modified texture diets, will have a major and potentially long-lasting impact on the person’s lifestyle as well as health.

There are few direct reports on how informed consent for modified texture diets is obtained in routine clinical practice. The evidence we do have, including surveys of practitioners and patients in different settings, provides ample reason to suspect that current practice is often inadequate. Sometimes there may be limited communication—and no real attempt to obtain consent—before starting modified texture diets. Even where the need for informed consent is recognized recommendations regarding use of modified texture diets may be “commonly influenced by myths, misconceptions, fear, and cognitive biases” [18, p. 953]. Such misconceptions seep into informed consent discussions and ‘poison the well’ for all stages of the process:

- Patients cannot make appropriate decisions for themselves if the information that they are given by professionals is flawed and unbalanced.
- The voluntariness of patients’ decisions is questionable if they are told ‘you must’ when ‘you might consider’ is more appropriate.
- Unless based on accurate and balanced information, inappropriate judgements and recommendations may be made where the decision-making capacity of patients is in question.

There are a number of steps we believe necessary to ensure that valid informed consent is sought for modified texture diets:

- Research is required to examine in greater detail current informed consent processes in different settings.
- There is a need for staff to reflect on their current practice regarding use of modified texture diets with an awareness of the currently available evidence and through the ‘lens’ of informed consent, and there is a need for education of staff about the importance of, and requirements for, valid informed consent and true shared decision making.
- A significant change in practice is needed in those residential care settings where there is insufficient access to SLTs and where staff may recommend or even impose modified texture diets without an adequate understanding of the issues involved. Not only is informed consent impossible in these circumstances, this approach represents poor practice.
- Professional bodies and guidelines regarding management of dysphagia should be clear about the need for informed consent before use of modified texture diets.

## Data Availability

Not applicable.

## Abbreviations

SDM: Shared decision making
SLT: Speech and language therapists
TL: Thickened liquids
MF: Modified foods
QOL: Quality of life
MTDs: Modified texture diets
